# Rare causes of pediatric anaphylaxis due to obscure allergens

**DOI:** 10.3389/falgy.2024.1456100

**Published:** 2024-08-26

**Authors:** Shajitha Melethil, Ejaz Yousef

**Affiliations:** Division of Allergy and Immunology, Nemours Children’s Health, Jacksonville, FL, United States

**Keywords:** anaphylaxis, pediatric, rare, additives, excipients, obscure occult, allergen

## Abstract

This review provides a comprehensive overview of rare causes of pediatric anaphylaxis related to obscure allergens. Anaphylaxis, a severe hypersensitivity reaction, can occur without typical symptoms, posing diagnostic challenges, especially in children. Idiopathic anaphylaxis, where no trigger is identified despite thorough evaluation, is notably challenging in this population. This review synthesizes current literature, highlighting obscure triggers such as food additives, spices like fenugreek, and cross-reactive allergens, including lupine and gelatin. These allergens are often overlooked and can lead to misdiagnosis of idiopathic cases. Understanding these uncommon triggers is crucial for clinicians to ensure accurate diagnosis and effective management of pediatric anaphylaxis, emphasizing the need for heightened clinical awareness and further research. This review raises awareness among health care providers about these lesser-known causes, aiming to improve outcomes and quality of life for pediatric patients at risk of anaphylactic reactions.

## 1 Introduction

Anaphylaxis is a rapid-onset, severe systemic hypersensitivity reaction that can compromise the airway, breathing, and/or circulation, occurring with or without skin or cutaneous symptoms (1, 2). In children, common triggers include food, medications, and venom. Idiopathic anaphylaxis (IA) is diagnosed when no triggers are identified despite thorough evaluation (3–6). IA is more common in adults than in children; based on available data, 30%–60% of adult case of anaphylaxis are considered idiopathic, while approximately 10% of pediatric cases are considered idiopathic (7–9). In Asia-Pacific territories like Thailand, Singapore, Hong Kong, and China, 22% of pediatric cases were idiopathic (10). IA poses a significant diagnostic challenge and affects patients' quality of life. Hidden and uncommon allergens should be thoroughly considered before diagnosing IA (11–13). Currently, there is scarce information in medical literature about the rare causes in children. This review emphasizes rare causes of pediatric anaphylaxis, urging clinicians to remain vigilant.

## 2 Food and drug additives

Food additives can preserve, flavor, sweeten, thicken/emulsify, color, or stabilize food. These substances, either natural or synthetic, are not typical nutritive food ingredients and are rarely consumed alone. Up to 2% of children have reported adverse reactions to food additives, most of which are not likely immune-mediated, with higher rates (2%–7%) in those with atopic backgrounds (14–17). The U.S. Food and Drug Administration (FDA) has approved over 3,000 compounds as food additives and around 773 chemical agents for drug products (18, 19). U.S. and European Union (EU) regulations require the declaration of all intentionally added ingredients. However, certain composite foods like spices and natural or artificial flavors can be listed collectively, risking exposure to hidden allergens.

### 2.1 Spices

Allergy to spices is rare (2%–6.4% of total food allergies) and usually presents in adulthood (20).

Mustard is an important spice crop worldwide and belongs to the family Brassicaceae. Oral ingestion of mustard can trigger allergic reactions, including oral allergy syndrome, anaphylaxis, allergic rhinitis, asthma, and atopic dermatitis. In a study involving 49 mustard-allergic children in France, clinical manifestations included skin reactions (urticaria, angioedema) in 21 patients (42.8%), gastrointestinal reactions in 1 patient (2%), and conjunctivitis in 2 patients (4%) (21). Additionally, oral allergy syndrome and anaphylaxis were reported in 2% of the children (21).

There are very few studies that report angioedema and anaphylaxis after spice ingestion in children. Yazıcı et al. reported a case of a 13-year-old who developed angioedema upon exposure to three plants of the Lamiaceae family: *Salvia officinalis* (common sage), *Mentha piperita* (peppermint), and *Origanum onites* L (oregano) confirmed by oral food challenge (22).

Another case report discusses a 17-month-old child developing anaphylaxis after consuming venison seasoned with black pepper; skin and sIgE tests were positive for black pepper (23).

Another spice rarely reported as causing anaphylaxis in the pediatric age group is fenugreek. Fenugreek (*Trigonella foenum-graecum*) belongs to the Leguminosae family, along with other legumes like peanuts, soybeans, green peas, lentils, beans, chickpeas, and lupins. Fenugreek is an aromatic spice often used in Indian-style cooking. Faeste et al. found considerable homologous IgE-binding epitopes between fenugreek protein and major peanut allergens Arah1, Arah2, and Arah3, resulting in significant cross-reactivity (24). Che et al. reported a case of a 14-year-old with known allergies to peanuts, lentils, chickpeas, and peas who developed anaphylaxis to fenugreek after eating curry at a restaurant (25). They also reported a case of another 14-year-old with known food allergies to cashews who developed anaphylaxis possibly to sumac spice after eating a fattoush salad (25). Sumac (*Rhus coriaria* L.) and cashew both belong to the Anacardiaceae family, causing the possibility of cashew-sumac cross-reactivity resulting in anaphylaxis.

### 2.2 Food preservatives

Sulfites are used as food or drug additives for several different technical purposes, including to prevent enzymatic and non-enzymatic browning of fresh fruits and vegetables for their antioxidant properties and bleaching effects (19). Although sulfite-induced asthma has been reported in the pediatric age group, anaphylaxis is rare. There is a case report presented by Vitaliti et al. about a 5-year-old who developed anaphylaxis secondary to sodium metabisulphite sensitization (26). This child, who has a strong atopic background, reacted to multiple preserved foods and drugs, but skin prick tests for these foods and medications were negative (26). Diagnosis of sulfite allergy was established based on clinical history, specific IgE test for sulfite by ELISA immuno-enzymatic-reaction and patch test, as well as abatement of her symptoms following cessation of sulfite-containing foods and medications (26). Authors raised concerns regarding the treatment of patients who develop anaphylaxis to sulfites, which can be challenging since adrenaline and epinephrine products can contain sulfites, thus limiting treatment options (26).

### 2.3 Food additives

#### 2.3.1 Food coloring

There are no reported studies resulting in anaphylaxis due to food coloring in children. Leung et al. reported a case of a 17-year-old who developed anaphylaxis as well as biphasic reaction to patent blue vital dye (PBV), which is frequently used as a perioperative drug for lymphangiography (27). Interestingly, it also is used as a food additive. One case reported a 2-year-old with recurrent urticaria and angioedema upon exposure to annatto dye (28).

#### 2.3.2 Sugar alcohols

A double-blind placebo-controlled food challenge for an 11-year-old confirmed anaphylaxis to erythritol (29). The patient developed anaphylaxis after ingesting a diet sauce and health food containing erythritol (29).

Anaphylaxis to xylitol was reported in a 2-year-old with a history of allergy to cow's milk and hen's egg. This was confirmed by the skin prick test and basophil activation test (30).

#### 2.3.3 Food thickening agents/emulsifiers

Pectin is used as an emulsifier in jellies, jams, and candies, as a thickening agent in drinks, dessert fillings, and medicines, and as a source of dietary fiber. It is a structural heteropolysaccharide and shares common antigenic determinants with tree nuts like cashews and pistachios from the Anacardiaceae family. Pectin is commercially derived from apple or citrus fruits. Inhalation of pectin has been associated with rhinitis and occupational asthma, but there are few cases of IgE-mediated anaphylaxis in pediatric patients after ingestion of pectin. Pectin allergy should be considered in unexplained anaphylaxis, particularly in patients with cashew or pistachio allergies (31–34).

Gelatin is a partially denatured protein from animal collagen (bovine or porcine skin and bones). It is found in foods like gummy candies, medications like capsules or ointments, cosmetics, and vaccines (35). Though known to cause anaphylaxis, some patients do not consistently react to gelatin. Extensive hydrolyzation lowers its molecular weight and gel melting point, making it less allergenic. Thus, processing and dosing variability can affect its allergenicity (35, 36). Fish gelatin, prepared from fish like tuna, pollock, cod, and salmon, is available for dietary or religious reasons. There are few reports of fish gelatin causing anaphylaxis (37). Kuehn et al. reported an anaphylaxis case after ingestion of marshmallows with fish gelatin (38).

Fish gelatin may not be declared on allergy labels in the EU, as certain ingredients have temporary exemptions (39). With the use of fish gelatin-containing products expanding, it should be considered in children with idiopathic anaphylaxis, especially those with fish allergies.

#### 2.3.4 Lupine

Lupin, or lupini, is a legume of the genus Lupinus. Among the three Mediterranean species (blue, white, and yellow), white lupin is mainly used in the food industry, while yellow and blue lupin serve as livestock fodder. Lupin is versatile in food products, often added to milled flour to enhance flavor, color, and texture in baked goods, pasta, and vegan substitutes. It's also a growing plant-based protein source in gluten-free products.

Allergic reactions to lupin can be a primary lupin allergy or due to cross-reactivity with other legumes, especially peanuts. Reports in Europe indicate peanut-allergic patients reacting to lupin (40). In Canada, Soller et al. documented the first anaphylactic case in a 10-year-old who has allergies to peanuts and tree nuts, leading to a Health Canada advisory on lupin allergy (41). The U.S. Food Allergen Labeling and Consumer Protection Act does not require lupin labeling. Bingemann et al. noted an 11-year-old, with no prior food allergy, experiencing anaphylaxis from waffles containing lupine (42).

A French study by Muller et al. found that approximately two-thirds of peanut-allergic children were sensitized to other legumes, including lupin, and one-quarter were diagnosed with legume allergies (43). Half of the reactions were severe, including anaphylaxis. With the global rise in legume consumption, recognizing cross-allergies is crucial. In the United Kingdom and EU, only three legumes are priority allergens: peanut, soy, and lupin. Other legumes like pea protein and fenugreek also can cause severe allergic reactions.

### 2.4 Anisakis

Anisakiasis is a parasitic disease first described in the 1960s in the Netherlands. IgE-mediated allergy was reported in the 1990s (44). It is caused by the consumption of raw or undercooked seafood contaminated by third-stage larvae of the nematode Anisakidae family (*Anisakis simplex*, *A. pegreffii*, and *Pseudoterranova decipiens*). IgE-mediated allergy to *Anisakis simplex* has been described in Europe (especially Spain) and Asia (mainly Japan) (44–46). Food globalization has led to culinary diversity and an increase in the consumption of raw fish in different parts of the world, leading to reports of Anisakiasis from North and South America including the U.S. (47). Anisakis is a rare cause of anaphylaxis in the pediatric age group (45, 46). Centonze et al. reported the case of an 8-year-old who initially presented with anaphylaxis and progressively worsening right testicular pain (48). A histological exam confirmed extra-gastrointestinal anisakiasis (48). If there is clinical suspicion, a skin prick test and sIgE levels of the total extract or specific allergen components will help confirm the diagnosis.

### 2.5 Pancake syndrome

Oral mite anaphylaxis (OMA), or pancake syndrome, was first described by Erben et al. in 1993 (49). OMA can occur at any age, including in young children (50–54). It is characterized by anaphylaxis after consuming foods made with mite-contaminated flour, including wheat, oats, and corn flour. A recent update by Sánchez-Borges et al. reports over 200 OMA patients worldwide from countries such as Spain, Ireland, Belgium, the U.S., Venezuela, Peru, Panama, Japan, Singapore, Thailand, and Taiwan (55). The pathogenesis involves the rapid absorption of mite allergens in sensitized individuals. Various species of domestic and storage mites are responsible for OMA. Notably, approximately 40% of OMA patients also have non-steroidal anti-inflammatory drug (NSAID) hypersensitivity (56), and exercise-induced anaphylaxis has been reported with OMA (57, 58).

### 2.6 Lipid transfer protein syndrome

Nonspecific lipid transfer proteins (nsLTP) are heat-stable, pepsin digestion-resistant small proteins that are plant pan allergens (59). IgE-mediated reactions, including anaphylaxis to nsLTPs, have been reported in multiple studies in Southern European countries, but there is significant data emerging from other parts of the world (60). To date, there have been only three reported cases of pediatric lipid transfer protein (LTP) in the U.S (61). It frequently involves a co-factor for eliciting the reaction, involving exercise and NSAIDs. Fruits belonging to the Rosaceae family are the main culprits for nsLTP-induced anaphylaxis, especially peach (Pru p3), as per the European studies (60). However, a recent retrospective chart review conducted in Latin America found tree-nuts, like walnut and almond, trigger anaphylaxis instead of peach likely due to difference in dietary patterns (62). There also is an increasing body of evidence that suggests against primary sensitization to a pollen source (63).

Cabrera-Freitag et al. reported nsLTP-related anaphylaxis after passive exposure to cannabis in two adolescents, proposing that nsLTP Can s 3 involved in cannabis-fruit and vegetable syndrome as the culprit allergen (64).

## 3 Drug and vaccine excipients

Excipients and residues in drugs and vaccines can function as hidden allergens (65–67). Dextran, a vaccine stabilizer, caused hypersensitivity reactions post-MMR vaccination in Brazil (68). Rotarix, a live rotavirus vaccine, contains dextran. The yellow fever (YF) vaccine, grown in chicken eggs, may contain residual egg ovalbumin, potentially triggering severe reactions. Limited data exists on YF vaccine use in egg-allergic patients. Tanos Lopes et al. reported 435 children, of which approximately 33% had a possible history of egg anaphylaxis, 95.2% had no reactions, and of the 4.8% who had reactions, only one patient had possible anaphylaxis, implying the YF vaccine can be safely administered in egg-allergic children (69).

### 3.1 Carboxymethylcellulose

Carboxymethylcellulose (CMC) is an anionic hydrosoluble substance used in food and pharmaceutical industries as a stabilizing agent. IgE-mediated anaphylaxis has been reported in adults following intra-articular steroid injections and in barium sulfate suspensions used as contrast media. Pediatric anaphylaxis to CMC in drugs has not been reported, but there is a case of a 14-year-old who experienced anaphylaxis after consuming a CMC-containing ice lolly/popsicle (70).

### 3.2 Mannitol

In 1979, Lamb and Keogh first reported a case of anaphylactoid reaction to mannitol infusion in a 16-year-old with an atopic background (71). Lightner et al. reported a case of a 19-month-old who experienced anaphylaxis to mannitol (72). The child had a history of drug hypersensitivity and received mannitol for diuresis after chemotherapy (72). The authors concluded that patients with atopic backgrounds should be carefully monitored when receiving mannitol. Mannitol also has been used as a food additive (E421 as per European Directives on food labeling) and a low-calorie sweetener.

### 3.3 Polysorbate

Polysorbates, akin to Polyethylene glycol, are utilized in various injectable medications such as local anesthetics, glucocorticoids, biologics (e.g., omalizumab, adalimumab, ustekinumab), erythropoietin, darbepoetin, antibiotics, creams, and vaccines due to their emulsifying and stabilizing properties. Perino et al. documented a case involving a 15-year-old who experienced anaphylaxis after the initial omalizumab injection for asthma (73). The patient tested positive for omalizumab and polysorbate 20 (an excipient), suggesting polysorbate sensitization leading to omalizumab-induced anaphylaxis (73).

### 3.4 Polymyxin

Contact dermatitis has been reported after the use of topical antimicrobial medication, but anaphylaxis is extremely rare. Henao et al. reported a 2-year-old who developed anaphylaxis after administering polymyxin B-trimethoprim eye drops for the second time (74). IgE-mediated hypersensitivity to polymyxin was confirmed by skin testing, which included full-strength polymyxin, and was negative for the trimethoprim component.

Polymyxin also is used as a vaccine additive, so physicians must exercise caution if there is a reported history of sensitization.

### 3.5 2-Phenoxyethanol

Nakayama et al. reported a cluster of anaphylaxes in children following the administration of a trivalent split inactivated influenza vaccine (TIV) that contained 2-phenoxyethanol (2-PE) as the preservative in 2011–12 in Japan (75). IgE antibodies were detected against influenza vaccine materials, but the mechanisms that resulted in this influence of 2-PE are not yet clear. The incidence of anaphylaxis events reduced after 2-PE was changed to thimerosal in the 2012–13 season.

### 3.6 Polyethylene glycol

Type I hypersensitivity reactions to polyethylene glycol (PEG)/macrogls 3350 (76), specifically PEG-2000-N used in COVID-19 mRNA vaccines, have gained significant attention post-pandemic. PEG serves as an additive in medications (e.g., laxatives, steroids), chemotherapeutic agents for stabilization, and in food and cosmetics. Anaphylaxis to PEG in children is exceedingly rare. Khalid and Bundy reported an 11-year-old experiencing anaphylaxis to intravenous pegaspargase and oral Miralax (PEG-3350) (77). Hamano et al. documented a 3-year-old with anaphylaxis to oral olopatadine hydrochloride (PEG 4000) and oral xylocaine pump spray 8% (PEG 400) (78). The mechanism of PEG hypersensitivity, likely IgE-mediated Type I reactions, remains poorly understood. Issues such as inconsistent terminology, inadequate labeling, and gaps in knowledge contribute to potential misdiagnosis (79).

### 3.7 Human serum albumin

Adverse reactions to human serum albumin (HSA) are rare. Wang et al. reported a case of a 10-year-old who underwent plasmapheresis for Guillain-Barré syndrome and immediately developed anaphylaxis to the HSA in the replacement fluid (80). Basu et al. reported a case series, which includes a 15-year-old with anaphylaxis to 5% HSA (81). In both cases, titrated intra-dermal testing was employed to establish diagnosis as no standardized skin test was available. Basu et al. also proposed a diagnostic algorithm consisting of a histamine release test and titrated intravenous provocation if skin testing is equivocal (81). HSA hypersensitivity should be considered, especially when reactions happen in peri-operative settings or in relation to infusions. It could be seen as an occult allergen as it may not be the main ingredient in the formulation.

### 3.8 Airborne anaphylaxis

Both food and medications can trigger anaphylaxis via the inhaled route, albeit rare (82). Multiple foods have been reported to be elicitors, including cow's milk, legumes, seafood, grains like rice, buckwheat, seeds, and potatoes. Foods used as drug excipients, like lactose, also can trigger anaphylaxis in food-allergic individuals (83). Morikawa et al. reported on a 6-year-old with a cow milk allergy who developed anaphylaxis after inhaling laninamivir octanoate hydrate to treat influenza, which contained lactose as an excipient (84). Dry powder inhalers may contain lactose as excipients and precipitate anaphylaxis (85).

## 4 Uncommon food allergy syndromes causing anaphylaxis

### 4.1 Bird-egg syndrome

This is uncommon in children. In 1993, Añíbarro et al. described a 5-year-old child who developed asthma triggered by bird feathers and a year later developed anaphylaxis to egg which was the first reported case in the pediatric age group (86). Serum albumins are the highly cross-reacting allergens responsible for this syndrome. They can be found in bird feathers, muscle tissue and egg yolk (α-Livetin or Gal d 5) (87).

### 4.2 Pork-cat syndrome

This IgE-mediated hypersensitivity reaction stems from cross-reactivity between cat serum Albumin (Fel d 2) and porcine (Sus s 1) and beef (Bos d 6) serum albumins. Cases in children are rare (88–91). Symptoms typically occur post-consumption of raw or smoked meats, as pork serum albumin is heat-labile. Reduced exposure to cats may lower sIgE to cats, potentially improving tolerance to pork.

### 4.3 Alpha-gal syndrome

This allergic syndrome can lead to either immediate hypersensitivity to drugs containing alpha-gal (e.g., Cetuximab) or delayed hypersensitivity from consuming red meat from non-primate mammals (beef, pork, lamb). It typically follows a tick bite (*Amblyomma americanum*/lone star tick in North America, *Ixodes ricinus* in Europe, *Ixodes holocyclus* in Australia, and *Ixodes nipponensis* in Asia). The factors triggering alpha-gal IgE production post-tick bite remain unclear. Pediatric studies on alpha-gal syndrome (AGS) indicate lower incidence than in adults, lower sIgE levels to α-gal, mainly gastrointestinal symptoms, limited trigger foods, and relevance to cofactors like sports (92–95). AGS should be considered in the differential in children presenting with anaphylaxis in the absence of any apparent reason.

### 4.4 Exotic food anaphylaxis

Ballardini et al. reported a 13-year-old with a known allergy to chicken meat who presented with anaphylaxis after ingesting crocodile meat (96). It was hypothesized that IgE cross-reactivity between α-parvalbumins in chicken and crocodile meat was the culprit.

Anaphylaxis after ingestion of fish roe has been reported in a few pediatric cases and should be suspected in patients who develop anaphylaxis after consuming seafood but have absent seafood allergy (97–101).

### 4.5 Food-dependent exercise-induced anaphylaxis

Although it is a distinct clinical entity, clinicians often miss anaphylaxis triggered by physical activity, especially when it occurs in conjunction with hidden food ingredients such as wheat, celery, and cabbage consumed before exercise (102).

## 5 Conclusion

This review highlights the challenge of “idiopathic anaphylaxis” in children, emphasizing rare triggers like food additives, spices (e.g., fenugreek), and cross-reactive allergens (e.g., lupine, gelatin). Awareness of these obscure causes remains on a careful and detailed clinical history, potentially with the aid of food diaries, and then targeted investigations based on these findings. This is crucial for accurate diagnosis and effective management of pediatric anaphylaxis, emphasizing the need for heightened clinical awareness.
